# Preparation of PSA-DOX/ICG-Lip and Evaluation of Its Efficacy Against Cervical Cancer

**DOI:** 10.3390/pharmaceutics18040434

**Published:** 2026-03-31

**Authors:** Jingya Bai, Jiamin Huang, Qian Zhang, Wenjun Su, Xiaohui Tang, Mukadaisi Amuti, Guorui Zhu, Qi Shen, Jian Yang, Mei Wang

**Affiliations:** 1 School of Pharmacy, Xinjiang Medical University, Urumqi 830011, China; bai1876352066@163.com (J.B.); woahjmjy@163.com (J.H.); zhangqianxjmu@163.com (Q.Z.); 13629951195@163.com (W.S.); mkds@stu.xjmu.edu.cn (M.A.); 18599750060@163.com (G.Z.); sq001@xjmu.edu.cn (Q.S.); yj365@hotmail.com (J.Y.); 2 Science & Technology Innovation and Transformation Service Center of Xinjiang Medical University, Urumqi 830011, China; tangxh0429@163.com; 3 Xinjiang Key Laboratory of Natural Medicines Active Components and Drug Release Technology, Xinjiang Medical University, Urumqi 830011, China; 4 Engineering Research Center of Xinjiang and Central Asian Medicine Resources, Ministry of Education, Urumqi 830011, China

**Keywords:** polysialic acid, liposomes, doxorubicin, indocyanine green, cervical cancer

## Abstract

**Objectives**: To fabricate polysialic acid (PSA)-modified liposomes co-loaded with doxorubicin (DOX) and indocyanine green (ICG) for synergistic chemotherapy and photothermal therapy, and to enhance the anti-cervical cancer efficacy of liposomes via neutrophil targeting. **Methods**: PSA-DOX/ICG liposomes (PSA-DOX/ICG-Lip) were prepared by microfluidic technology. The physicochemical properties, including drug encapsulation efficiency (EE), loading capacity (LC), particle size, polydispersity index (PDI), zeta potential, and stability, were systematically characterized. The in vitro anti-tumor activity was evaluated using cellular uptake, apoptosis assays, reactive oxygen species (ROS) detection, and a cell scratch test in HeLa and C33a cells. The in vivo therapeutic efficacy was verified using a nude mouse xenograft model of cervical cancer combined with histopathological analysis. **Results**: Microfluidic preparation yielded PSA-DOX/ICG-Lip with favorable physicochemical properties: the EE and LC of DOX were 96.52 ± 0.43% and 8.70 ± 0.04%, respectively, while those of ICG were 90.72 ± 1.10% and 0.82 ± 0.02%. The average particle size was 92.68 ± 1.14 nm with a PDI of 0.04 and a zeta potential of −9.66 ± 0.46 mV. The liposomes maintained good stability in terms of EE, particle size, PDI, and zeta potential after 28 days of storage at 4 °C and room temperature, with PSA modification significantly reducing the drug leakage rate. In vitro drug release studies showed that 808 nm laser irradiation triggered a significant increase in drug release from the liposomes. ICG encapsulated in liposomes mediated localized photothermal heating, and PSA targeting precisely confined the therapeutic effect to the tumor site, minimizing damage to adjacent normal tissues. In vitro experiments demonstrated that PSA-DOX/ICG-Lip, combined with laser irradiation, significantly enhanced cellular uptake, elevated intracellular ROS levels, inhibited cancer cell migration, and induced apoptosis. In vivo studies confirmed that this formulation markedly suppressed tumor growth in nude mice, with a tumor inhibition rate of 81.5%, and exhibited good biocompatibility without obvious organ toxicity. **Conclusions**: The microfluidically prepared PSA-DOX/ICG-Lip possesses high drug encapsulation efficiency, uniform particle size, good stability and sustained drug release properties. It can efficiently convert light energy into thermal energy, target neutrophils to enhance the affinity for cervical cancer cells, and exert a synergistic anti-tumor effect via the combination of chemotherapy and photothermal therapy, which provides a promising nanoplatform for the precise treatment of cervical cancer.

## 1. Introduction

Cervical cancer is one of the most prevalent gynecological malignancies worldwide, ranking fourth in both incidence and mortality among all female cancers. In 2020, there were approximately 604,000 new cases and 342,000 cancer-related deaths from cervical cancer globally, with China accounting for 18.3% (110,000 cases) and 17.6% (47,700 deaths) of the global totals, respectively [1]. Although the human papillomavirus (HPV) vaccine has become an effective preventive measure, the high incidence and mortality of cervical cancer persist in developing countries due to inadequate medical resources and insufficient public health intervention strategies. Thus, the development of more effective and safe therapeutic strategies for cervical cancer is an urgent clinical need.

**Figure 1:**
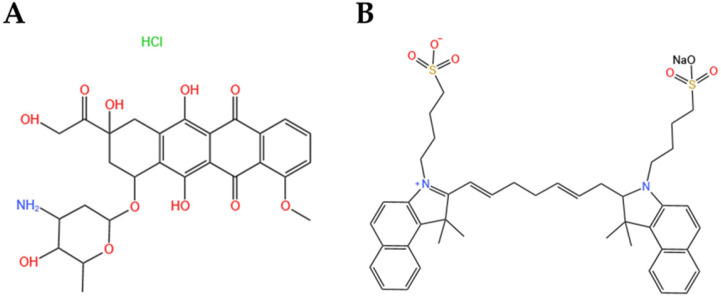
(**A**) shows the chemical structure of doxorubicin hydrochloride (DOX); (**B**) shows the chemical structure of ICG.

Traditional therapeutic modalities for cervical cancer include surgery, radiotherapy, and chemotherapy; yet these approaches are limited by severe off-target side effects, high recurrence rates, and poor patient compliance [2]. In recent years, targeted therapy and immunotherapy have emerged as promising alternatives for cancer treatment, which improve therapeutic efficacy by precisely targeting tumor cells or the tumor microenvironment (TME) while reducing damage to normal tissues [3]. As a classic anthracycline chemotherapeutic drug, doxorubicin (DOX) exerts anti-tumor activity by intercalating into DNA double strands and inhibiting topoisomerase II (Figure 1A), thereby blocking DNA replication and transcription in rapidly proliferating tumor cells. However, the clinical application of free DOX is severely restricted by its cardiotoxicity, myelosuppression, poor water solubility, and non-specific tissue distribution. Photothermal therapy (PTT), a non-invasive therapeutic approach, converts light energy into thermal energy via photothermal agents to induce tumor cell necrosis or apoptosis, and has attracted extensive attention for its high spatial-temporal selectivity and minimal side effects. Indocyanine green (ICG), a clinically approved near-infrared (NIR) fluorescent dye, is a promising photothermal agent with good biocompatibility, strong NIR light absorption, and efficient photothermal conversion efficiency (Figure 1B). Nevertheless, the clinical translation of free ICG is hindered by its inherent instability, rapid clearance, and potential phototoxicity to normal tissues caused by non-specific distribution. Consequently, the single application of PTT is limited by insufficient tumor targeting and poor therapeutic efficacy for deep-seated tumors, while the combination of chemotherapy and PTT can achieve synergistic anti-tumor effects and overcome the limitations of monotherapy.

Liposomes, as classic nano-drug delivery systems, have been widely applied in cancer therapy due to their excellent biocompatibility, low immunogenicity, biodegradability, and tunable surface modification [4]. Liposomal encapsulation can improve the solubility and bioavailability of chemotherapeutic drugs, prolong their in vivo circulation time, and reduce off-target side effects [5]. However, unmodified liposomes face challenges such as poor tumor targeting, easy clearance by the mononuclear phagocyte system (MPS), and limited penetration of the biological barrier. PEGylation is a common surface modification strategy to prolong the in vivo circulation of liposomes, but it may induce immune responses and reduce the cellular internalization efficiency of liposomes [6,7]. Thus, developing novel surface modification strategies to enhance tumor targeting and therapeutic efficacy of liposomes is of great significance.

The TME is a complex microenvironment composed of tumor cells, immune cells, stromal cells, and extracellular matrix. Neutrophils are the most abundant innate immune cells in peripheral blood, accounting for 50–70% of circulating white blood cells [8,9]. Tumor-associated neutrophils (TANs) are a subgroup of neutrophils recruited to the TME, which can be polarized into the anti-tumor N1 phenotype and the pro-tumor N_2_ phenotype with plastic phenotypic and functional characteristics [10,11,12,13]. Pro-tumor N_2_ neutrophils participate in all stages of tumor progression, including tumorigenesis, metastasis, and immunosuppression, and are actively recruited to the TME by cytokines secreted by tumor cells [14,15]. L-selectin [16], a cell surface adhesion molecule highly expressed on neutrophils, mediates neutrophil adhesion, migration, and activation, and is a potential target for neutrophil-based tumor targeting [17,18]. Polysialic acid (PSA), a hydrophilic linear polysaccharide composed of N-acetylneuraminic acid (Neu5Ac) units, is the natural ligand of L-selectin [19]. PSA with α-2,8 glycosidic linkages is a non-immunogenic, biodegradable endogenous polymer that can be degraded into non-toxic sialic acid by tissue sialidases [20]. Compared with PEG, PSA not only has similar hydrophilicity, flexibility, and controllable molecular weight, but also can achieve active targeting to neutrophils via the specific binding of PSA and L-selectin, improve the enhanced permeability and retention (EPR) effect of nano-drugs in tumor tissues, and reduce MPS-mediated clearance [21]. Therefore, PSA modification is an ideal strategy to enhance the tumor targeting and in vivo stability of liposomes.

Microfluidic technology, a novel nanomaterial preparation method, has significant advantages over traditional liposome preparation methods (e.g., thin-film hydration, reverse evaporation). It enables continuous and large-scale preparation of liposomes by precisely manipulating microscale fluid flow, and can accurately control the physicochemical properties of liposomes (e.g., particle size, PDI, drug loading) with high batch-to-batch reproducibility. In addition, microfluidic technology has the characteristics of low reagent consumption, high production efficiency, and rapid heat transfer, which are suitable for the preparation of functional nano-drug delivery systems.

In this study, we prepared PSA-modified DOX/ICG co-loaded liposomes (PSA-DOX/ICG-Lip) by microfluidic technology for synergistic chemotherapy and PTT of cervical cancer. The physicochemical properties, stability, photothermal conversion efficiency, and pH/laser dual-responsive drug release behavior of the liposomes were systematically characterized. The in vitro anti-tumor effects (cellular uptake, ROS generation, anti-proliferation, anti-migration) were evaluated on human cervical cancer HeLa and C33a cells, and the in vivo anti-tumor efficacy and biosafety were verified using a nude mouse xenograft model of cervical cancer. We hypothesized that PSA modification could achieve active targeting of liposomes to neutrophils and tumor cells, and the combination of DOX-mediated chemotherapy and ICG-mediated PTT could exert synergistic anti-tumor effects, providing a novel and effective nano-therapeutic strategy for cervical cancer.

## 2. Materials and Methods

### 2.1. Materials

Heating magnetic stirrer (RCT Basic, IKA Corporation, Staufen, Germany); electronic balance (BP211D, Sartorius Group, Shanghai, China); heated numerical control ultrasonic cleaner (KH-300DE, Kunshan Hechuang Ultrasonic Instrument Co., Ltd., Kunshan, Jiangsu, China); constant temperature shaker (Jiangsu Jintan Medical Instrument, Changzhou, Jiangsu, China); ultrasonic cleaner (SY-360, Beijing Tianpeng Electronic New Technology Co., Ltd., Beijing, China); laser particle size and zeta potential analyzer (ZEN3600, Malvern Company, Malvern, UK); PSA (Zhongke Hongji Biotechnology Co., Ltd., Jinzhong, Shanxi, China); DOX (Shanghai Macklin Biochemical Technology Co., Ltd., Shanghai, China); ICG (Bide Pharmatech Co., Ltd., Shanghai, China); dipalmitoyl phosphatidylcholine (DPPC), hydrogenated soy phospholipids (HSPC), cholesterol, and DSPE-PEG_2000_-COOH were purchased from AVT Pharmaceutical Tech Co., Ltd. (Shanghai, China); DMEM medium was purchased from Gibco Life Technologies (New York, NY, USA); the CCK-8 assay kit was purchased from Life-iLab Biotechnology Co., Ltd. (Shanghai, China); the Calcein-AMPI live/dead cell dual staining kit was purchased from Solarbio (Beijing, China); the ROS assay kit and DCFH-DA was purchased from Beyotime Biotechnology Co., Ltd. (Shanghai, China); and the neutrophil isolation solution was purchased from Haoyang Biological Manufacture Co., Ltd. (Tianjin, China); syringe pump (Jiangsu Zhiyu Medical Instrument Co., Ltd., Taixing, Jiangsu, China); transmission electron microscope HT7800 (HITACHI, Tokyo, Japan); carbon-coated copper grid (Zhongjing Scientific Instruments, Beijing, China); dialysis bag (Solarbio, Beijing, China); UV-visible spectrophotometry 2700 (SHIMADZU, Kyoto, Japan); constant-temperature shaker (Shanghai Bosun Medical and Biological Instruments Co., Ltd., Shanghai, China); NIR laser (NBeT Group Corp., Beijing, China); thermal imaging camera 323pro (Fotric Inc., Shanghai, China); LCSM ECLipSE Ti (NIKON, Shanghai, China); fluorescence inverted microscope DMi8 (LEICA, Wetzlar, Germany); Human cervical cancer HeLa cells were obtained from the Wuhan Servicebio Technology Co., Ltd. (Wuhan, China); C33a cells were obtained from the laboratory (Xinjiang, China); BALB/c-nu nude mice (female, 4–6 weeks old, 18–22 g) were purchased from Beijing Vital River Laboratory Animal Technology Co., Ltd. (Beijing, China)

### 2.2. Preparation of PSA-DOX/ICG-Lip

PSA-DOX/ICG-Lip was prepared by microfluidic technology combined with the ammonium sulfate gradient method. Briefly, DPPC, HSPC, cholesterol, and DSPE-PEG_2000_-PSA were mixed at a molar ratio of 3:3:2:0.5 and dissolved in methanol to form the organic phase (Solution A). ICG was dissolved in 0.25 M ammonium sulfate solution to form the aqueous phase (Solution B). Solution A and Solution B were simultaneously injected into a microfluidic mixer using a syringe pump at a total flow rate of 12 mL/min and an organic/aqueous phase flow rate ratio of 1:2. The obtained liposome suspension was dialyzed against phosphate-buffered saline (PBS) (pH 7.4) for 4 h to remove methanol and establish the ammonium sulfate gradient. DOX solution was added to the liposome suspension at a drug-lipid mass ratio of 1:10, and the mixture was incubated at 60 °C for 30 min to load DOX into the liposomes via the ammonium sulfate gradient method. The unencapsulated free DOX was removed by dialysis against PBS (pH 7.4) for 10 h, and the final PSA-DOX/ICG-Lip was obtained and stored at 4 °C in the dark. For comparison, blank liposomes (Blank-Lip), DOX-loaded liposomes (DOX-Lip), ICG-loaded liposomes (ICG-Lip), and unmodified DOX/ICG co-loaded liposomes (DOX/ICG-Lip) were prepared using the same method without adding DSPE-PEG_2000_-PSA or the corresponding drugs.

### 2.3. Physicochemical Properties of Liposomes

#### 2.3.1. Morphological Characterization

The morphology of PSA-DOX/ICG-Lip was observed by transmission electron microscope (TEM). A 10 μL aliquot of the liposome suspension (diluted 10-fold with deionized water) was placed onto a carbon-coated copper grid, air-dried at room temperature, and stained with 10% phosphotungstic acid for 10 min. The excess staining solution was blotted with filter paper, and the morphology of the liposomes was observed under TEM at an acceleration voltage of 80 kV.

#### 2.3.2. Particle Size, PDI, and Zeta Potential

The liposome suspension was diluted 10-fold with deionized water, and the particle size, PDI, and zeta potential were measured using a laser particle size and zeta potential analyzer at 25 °C. Each sample was tested in triplicate, and the average value was calculated.

#### 2.3.3. Encapsulation Efficiency (EE) and Drug Loading Capacity (LC)

The EE and LC of DOX and ICG in the liposomes were determined by the dialysis method combined with UV-visible spectrophotometry. Briefly, 1 mL of the liposome suspension was placed in a dialysis bag (MWCO: 8000 Da) and dialyzed against 50 mL of PBS (pH 7.4) for 24 h at 37 °C with gentle shaking to remove unencapsulated drugs. After dialysis, the liposome suspension in the dialysis bag was disrupted with methanol (liposome/methanol volume ratio = 1:9), and the concentration of DOX and ICG was measured by UV-visible spectrophotometry at 480 nm and 780 nm, respectively. For the determination of the total drug amount, 1 mL of the liposome suspension was directly disrupted with methanol, and the drug concentration was measured using the same method. The EE and LC were calculated according to the following formulas:

EE (%) = W_1_/W_2_ × 100% (1)

LC (%) = W_1_/W_3_ × 100% (2)

In the formulas, W_1_ represents the amount of drug encapsulated in the liposomes after dialysis, W_2_ represents the total amount of drug initially added before dialysis, and W_3_ represents the total amount of liposomal membrane material and drug.

#### 2.3.4. In Vitro Drug Release

The pH/laser dual-responsive drug release behavior of DOX from the liposomes was evaluated by the dialysis method. Briefly, 3 mL of Free DOX, DOX/ICG-Lip, and PSA-DOX/ICG-Lip suspensions (DOX concentration: 100 μg/mL) were placed in dialysis bags (MWCO: 8000 Da), and immersed in 30 mL of release medium (PBS containing 0.5% (*w*/*v*) Tween 80, pH 7.4 or pH 5.0 (10 mM citrate)). The release system was incubated in a constant-temperature shaker at 37 °C and 200 rpm. For the laser irradiation group, the release system was irradiated with an 808 nm NIR laser (power density: 1 W/cm^2^) for 10 min at the beginning of the experiment, while the non-irradiation group was incubated in the dark. At predetermined time points (0.5, 1, 2, 3, 4, 5, 8, 12, 24, 36, 48 h), 1 mL of the release medium was collected, and an equal volume of fresh pre-warmed release medium was replenished. The concentration of DOX in the collected medium was measured by UV-visible spectrophotometry at 480 nm, and the cumulative drug release rate was calculated. Each sample was tested in triplicate.

#### 2.3.5. Stability Evaluation

The stability of the liposomes was evaluated by monitoring the changes in particle size, PDI, zeta potential, and drug leakage rate during storage. Blank-Lip, DOX-Lip, ICG-Lip, DOX/ICG-Lip, and PSA-DOX/ICG-Lip were stored at 4 °C and 25 °C in the dark for 28 days. At predetermined time points (1, 3, 5, 7, 14, 21, 28 days), the particle size, PDI, and zeta potential of the liposomes were measured, and the drug leakage rate was calculated by determining the concentration of the encapsulated drug using the method described in Section 2.3.3.

#### 2.3.6. Photothermal Property Evaluation

The photothermal conversion efficiency and photothermal stability of the liposomes were evaluated using an 808 nm NIR laser and a thermal imaging camera. (1) Optimal ICG concentration screening: Free ICG solutions with concentrations of 5, 10, 15, 20, 25, and 30 μg/mL were prepared, and irradiated with an 808 nm laser (power density: 0.8 W/cm^2^) for 10 min. The temperature of the solution was recorded every 30 s using a thermometer, and the temperature change curve was plotted. (2) Optimal laser power density screening: DOX/ICG-Lip suspension (ICG concentration: 15 μg/mL) was irradiated with 808 nm lasers at power densities of 0.2, 0.4, 0.6, 0.8 and 1.0 W/cm^2^ for 10 min, and the temperature change was recorded every 30 s. (3) Photothermal conversion efficiency of PSA-DOX/ICG-Lip: PSA-DOX/ICG-Lip suspensions with ICG concentrations of 10 and 15 μg/mL were irradiated with 808 nm lasers at power densities of 0.8 and 1.0 W/cm^2^ for 10 min, and the temperature change was recorded. (4) Comparison of photothermal properties: Free ICG, ICG-Lip, DOX/ICG-Lip, and PSA-DOX/ICG-Lip suspensions (ICG concentration: 15 μg/mL) were irradiated with an 808 nm laser (power density: 1 W/cm^2^) for 10 min, and the temperature change and thermal images were recorded. (5) Photothermal stability: DOX/ICG-Lip and PSA-DOX/ICG-Lip suspensions (ICG concentration: 15 μg/mL) were subjected to five freeze–thaw cycles (−20 °C/37 °C), and then irradiated with an 808 nm laser (power density: 1 W/cm^2^) for 10 min. The temperature change was recorded to evaluate the photothermal stability. (6) Thermal imaging analysis: ICG-Lip, DOX/ICG-Lip, and PSA-DOX/ICG-Lip suspensions (ICG concentration: 10 μg/mL) were irradiated with an 808 nm laser (power density: 1 W/cm^2^) for 15 min, and thermal images were captured every 30 s using a thermal imaging camera [22].

### 2.4. In Vitro Cell Studies

#### 2.4.1. Cytotoxicity Test

The cytotoxicity of the liposomes on HeLa and C33a cells was evaluated by the CCK-8 assay. HeLa cells (5 × 10^3^ cells/well) and C33a cells (8 × 10^3^ cells/well) were seeded into 96-well plates and cultured for 24 h to allow cell adhesion. The culture medium was replaced with fresh medium containing Free DOX, DOX-Lip, DOX/ICG-Lip, and PSA-DOX/ICG-Lip at different DOX concentrations (HeLa: 0, 0.312, 0.625, 1.25, 2.5, 5, 10, 20, 45, 90 μM; C33a: 0, 0.05, 0.1, 0.2, 0.4, 0.8, 1.6, 3.2, 6.4, 12.8 μM). The laser irradiation group was irradiated with an 808 nm laser (power density: 1 W/cm^2^) for 10 min after drug addition, while the non-irradiation group was incubated in the dark. After incubation for 48 h (HeLa) and 24 h (C33a), 10 μL of CCK-8 solution was added to each well, and the plates were incubated for an additional 40 min (HeLa) and 90 min (C33a). The absorbance at 450 nm was measured using a microplate reader.

#### 2.4.2. Calcein-AM/PI Live/Dead Cell Staining

The anti-proliferative effect of the liposomes on HeLa cells was intuitively evaluated by Calcein-AM/PI double staining. HeLa cells (5 × 10^4^ cells/well) were seeded into 6-well plates and cultured for 24 h. The culture medium was replaced with fresh medium containing Free DOX, DOX-Lip, DOX/ICG-Lip and PSA-DOX/ICG-Lip (DOX concentration: 5 μM). The laser irradiation group was irradiated with an 808 nm laser (power density: 1 W/cm^2^) for 10 min, and the non-irradiation group was incubated in the dark. After incubation for 48 h, the culture medium was discarded, and the cells were stained with Calcein-AM/PI staining solution (Calcein-AM: 2 μM, PI: 5 μM) for 30 min in the dark. The stained cells were observed and photographed under a fluorescence inverted microscope, where live cells emit green fluorescence and dead cells emit red fluorescence [23].

#### 2.4.3. Laser Confocal Scanning Microscopy (LCSM) for Cellular Uptake

The cellular uptake efficiency of the liposomes by HeLa and C33a cells was evaluated by LCSM. HeLa cells (2.5 × 10^5^ cells/dish) and C33a cells (5 × 10^5^ cells/dish) were seeded into confocal culture dishes and cultured for 12 h. The culture medium was replaced with fresh medium containing Free DOX, DOX-Lip, DOX/ICG-Lip, and PSA-DOX/ICG-Lip (DOX concentration: 5 μM), and the cells were incubated for 3 h. The cells were washed three times with cold PBS, fixed with 4% paraformaldehyde for 15 min, and stained with 4′,6-Diamidino-2′-Phenylindole (DAPI) (2 μg/mL) for 10 min to label the cell nuclei. The cellular uptake of the liposomes was observed and photographed under LCSM, where DOX emits red fluorescence, ICG emits near-infrared fluorescence, and DAPI emits blue fluorescence. The fluorescence intensity was analyzed using ImageJ 1.54f software.

#### 2.4.4. Flow Cytometry for Neutrophil Targeting

Neutrophils were isolated from the orbital blood of healthy BALB/c-nu mice using a Neutrophil Isolation Kit according to the manufacturer’s instructions, and the purity of neutrophils was identified by flow cytometry using Ly6g and CD11b as specific markers. HeLa cells, C33a cells, and isolated neutrophils (5 × 10^4^ cells/well) were seeded into 6-well plates and cultured for 24 h. PSA-DOX/ICG-Lip suspension (DOX concentration: 5 μM) was added to each well, and the cells were incubated for 3 h. The cells were digested with trypsin (for adherent cells) or collected by centrifugation (for neutrophils), washed three times with cold PBS, and resuspended in 500 μL of cold PBS. The fluorescence intensity of DOX in the cells was detected by flow cytometry, and the targeting ability of PSA-DOX/ICG-Lip to neutrophils was evaluated by comparing the fluorescence intensity in different cells.

#### 2.4.5. ROS Assay

The intracellular ROS generation induced by the liposomes was evaluated using the 2′,7′ -Dichlorofluorescein Diacetate (DCFH-DA) ROS Assay Kit. HeLa cells (2.5 × 10^5^ cells/well) and C33a cells (3 × 10^4^ cells/well) were seeded into 12-well plates and cultured for 24 h. The culture medium was replaced with fresh medium containing Free DOX, DOX-Lip, DOX/ICG-Lip, and PSA-DOX/ICG-Lip (DOX concentration: 5 μM). The laser irradiation group was irradiated with an 808 nm laser (power density: 1 W/cm^2^) for 10 min, and the non-irradiation group was incubated in the dark. After incubation for 3 h, the cells were washed three times with cold PBS and incubated with DCFH-DA probe (10 μM) for 20 min in the dark. The cells were washed again with cold PBS, stained with DAPI (2 μg/mL) for 10 min, and observed and photographed under a fluorescence inverted microscope. The fluorescence intensity of DCF (oxidized form of DCFH-DA) was analyzed using ImageJ 1.54f software to evaluate the intracellular ROS level.

#### 2.4.6. Cell Scratch Assay for Anti-Migration Effect

The anti-migration effect of the liposomes on HeLa and C33a cells was evaluated by the cell scratch assay. HeLa cells (2.5 × 10^5^ cells/well) and C33a cells (3 × 10^5^ cells/well) were seeded into 6-well plates and cultured until the cell confluency reached 90–100%. A straight scratch was made on the cell monolayer using a 200 μL pipette tip, and the detached cells were washed away with cold PBS. The culture medium was replaced with fresh serum-free medium containing Free DOX, DOX-Lip, DOX/ICG-Lip, and PSA-DOX/ICG-Lip (DOX concentration: 5 μM). The laser irradiation group was irradiated with an 808 nm laser (power density: 1 W/cm^2^) for 10 min, and the non-irradiation group was incubated in the dark. The scratch area was photographed under a fluorescence inverted microscope at predetermined time points (0, 6, 12, 24 h) [24].

### 2.5. In Vivo Anti-Tumor Efficacy Evaluation

#### 2.5.1. Establishment of Cervical Cancer Xenograft Mode

BALB/c-nu nude mice were acclimatized in a specific pathogen-free (SPF) animal facility for 1 week before the experiment. HeLa cells in the logarithmic growth phase were digested with 0.25% trypsin, resuspended in serum-free RPMI 1640 medium, and the cell concentration was adjusted to 1 × 10^7^ cells/mL. A 100 μL aliquot of the cell suspension was subcutaneously injected into the right axilla of each nude mouse. The tumor volume was measured every 2 days using a digital vernier caliper, and the tumor volume was calculated according to the formula V = ab^2^/2 (where a is the longest diameter and b is the shortest diameter perpendicular to a).

#### 2.5.2. In Vivo Administration and Therapeutic Efficacy

The mice in each group were intravenously injected with the corresponding formulations via the tail vein every other day for a total of 14 days (DOX dosage: 1 mg/kg). For the PSA-DOX/ICG-Lip + laser group, the tumor site was irradiated with an 808 nm NIR laser (power density: 1 W/cm^2^, irradiation time: 10 min) at 4 h after each drug injection. The body weight and tumor volume of the mice were measured every 2 days during the experiment.

#### 2.5.3. Hematoxylin–Eosin (H&E) Staining

The harvested tumors and major organs were fixed with 4% paraformaldehyde for 24 h, dehydrated with gradient ethanol, embedded in paraffin, and sectioned into 5 μm thick slices. The slices were stained with hematoxylin and eosin, and observed and photographed under an fluorescence inverted microscope to evaluate the tumor tissue necrosis and organ histopathological changes.

### 2.6. Statistical Analysis

All experimental data were expressed as the mean ± standard deviation (mean ± SD). Statistical analysis was performed using IBM SPSS Statistics 26.0 software and GraphPad Prism 10.1.2 software. One-way analysis of variance (ANOVA) followed by LSD post hoc test, Tukey’s HSD test, and Waller–Duncan multiple range test was used for multiple comparisons. Homogeneity of variance was tested before ANOVA. A *p*-value < 0.05 was considered statistically significant, and the statistical symbols were indicated as follows: * *p* < 0.05, ** *p* < 0.01, *** *p* < 0.001, **** *p* < 0.0001.

## 3. Results

### 3.1. Physicochemical Properties of Different Liposomes

**Figure 2:**
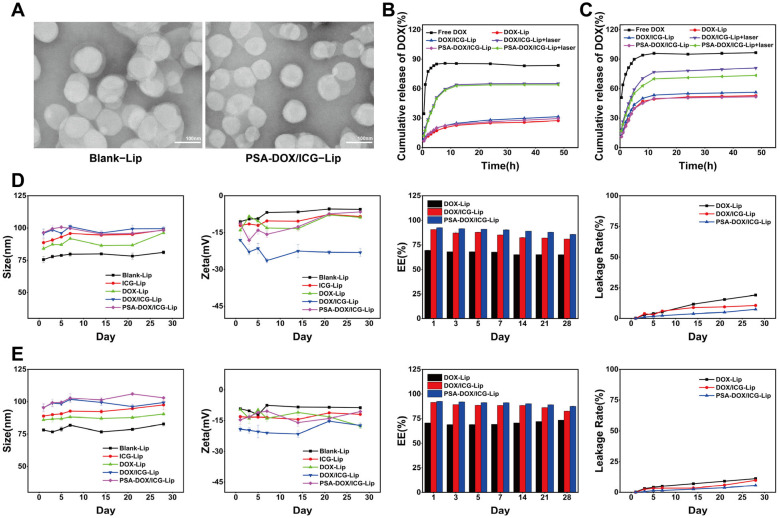
Physicochemical Properties of Different Liposomes. (**A**) TEM of liposomes; (**B**) Cumulative release of DOX from different liposomes in PBS; (**C**) Cumulative release of DOX from different liposomes in Citric acid; (**D**) Stability of different liposomes at 25 °C (*n* = 3, mean ± SD); (**E**) Stability of different liposomes at 4 °C (*n* = 3, mean ± SD).

The EE and LC of DOX and ICG in different liposomal formulations are summarized in Table 1. PSA-DOX/ICG-Lip exhibited the highest EE and LC among all formulations, with the EE of DOX and ICG reaching 96.52 ± 0.43% and 90.72 ± 1.10%, and the LC of 8.70 ± 0.04% and 0.82 ± 0.02%, respectively. The particle size, PDI, and zeta potential of different liposomes are shown in Table 2. PSA-DOX/ICG-Lip had a uniform particle size of 92.68 ± 1.14 nm, a low PDI of 0.04, and a negative zeta potential of −9.66 ± 0.46 mV, indicating good monodispersity and colloidal stability. TEM images (Figure 2A) showed that PSA-DOX/ICG-Lip was spherical in shape with a uniform particle size of approximately 90 nm, which was consistent with the dynamic light scattering (DLS) results.

The in vitro drug release behavior of DOX from different liposomal formulations in pH 7.4 PBS and pH 5.0 citrate buffer (mimicking the acidic TME) with or without 808 nm laser irradiation is shown in Figure 2B,C. Free DOX exhibited a rapid release profile, with nearly complete release within 8 h in both release media. In the non-irradiation group, the cumulative release rate of DOX from DOX/ICG-Lip and PSA-DOX/ICG-Lip was approximately 30–35% at 48 h in pH 7.4 PBS and 50–55% in pH 5.0 citrate buffer, indicating a pH-responsive drug release property. In the laser irradiation group (808 nm, 1 W/cm^2^), the cumulative release rate of DOX from DOX/ICG-Lip and PSA-DOX/ICG-Lip was significantly increased to approximately 65% and 63% in pH 7.4 PBS, and 80% and 75% in pH 5.0 citrate buffer at 48 h, respectively. The results indicated that the liposomes had a pH/laser dual-responsive drug release behavior, and the combination of acidic pH and laser irradiation could significantly promote drug release, which was beneficial for the targeted drug release in the acidic TME and for enhanced anti-tumor efficacy.

The stability of different liposomal formulations stored at 4 °C and 25 °C in the dark for 28 days is shown in Figure 2D,E. All liposomal formulations maintained a clear and homogeneous appearance without aggregation or precipitation for the first 21 days of storage at both temperatures, and slight aggregation was only observed on day 28. The particle size, PDI, and zeta potential of the liposomes showed no significant changes during storage (*p* > 0.05). The drug leakage rate of the liposomes increased with the extension of storage time, and the leakage rate at 25 °C was higher than that at 4 °C. Among all formulations, PSA-DOX/ICG-Lip exhibited the lowest drug leakage rate, with the leakage rate of DOX less than 8% at 4 °C and less than 10% at 25 °C after 28 days of storage. The excellent stability of PSA-DOX/ICG-Lip was attributed to the hydration layer formed by PSA on the liposome surface, which reduced the aggregation and fusion between liposomes [25].

### 3.2. In Vitro Drug Release Behavior

**Figure 3:**
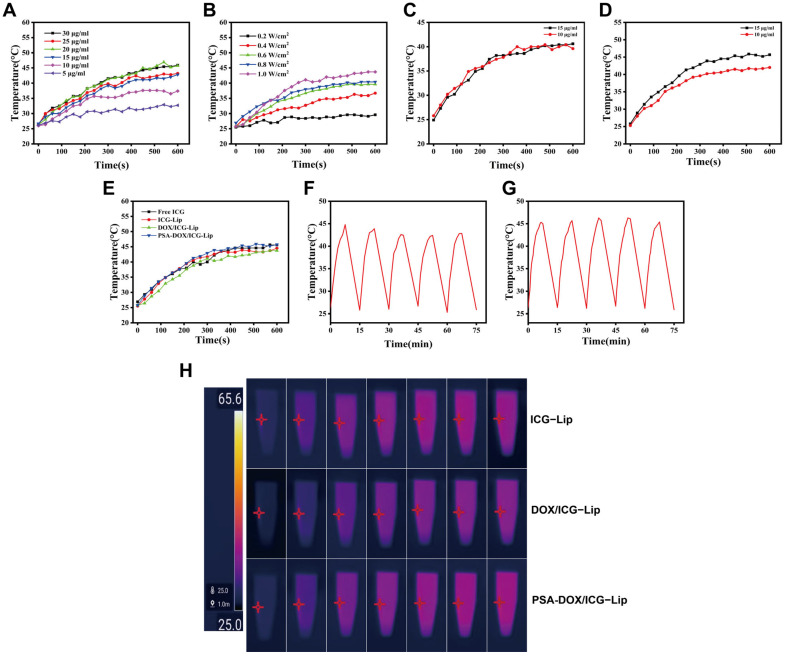
Photothermal Properties of Liposomes. (**A**) Photothermal conversion efficiency of Free ICG at different concentrations under laser irradiation (808 nm, 0.8 W/cm^2^); (**B**) Photothermal conversion efficiency of DOX/ICG-Lip (15 μg/mL) under irradiation by lasers of different powers; (**C**) Photothermal conversion efficiency of PSA-DOX/ICG-Lip at different concentrations upon laser irradiation (808 nm, 0.8 W/cm^2^); (**D**) Photothermal conversion efficiency of PSA-DOX/ICG-Lip at different concentrations upon laser irradiation (808 nm, 1 W/cm^2^); (**E**) Photothermal conversion efficiency of different liposomes; (**F**) Plot of temperature variation during hot and cold cycling of DOX/ICG-Lip; (**G**) Plot of temperature variation during hot and cold cycling of PSA-DOX/ICG-Lip; (**H**) Thermal imaging of different liposomes (ICG concentration: 10 μg/mL), the red asterisks in the image indicate the camera’s focus points.

The photothermal conversion properties of the free ICG and liposomal formulations were systematically evaluated, and the results are shown in Figure 3. The temperature of Free ICG solution increased with the increase in ICG concentration and laser power density (Figure 3A,B). Free ICG reached the photothermal therapeutic temperature (≥42 °C) at concentrations ≥ 15 μg/mL under 808 nm laser irradiation (0.8 W/cm^2^) for 10 min, and DOX/ICG-Lip reached the therapeutic temperature at a laser power density of 1 W/cm^2^ (ICG concentration: 15 μg/mL). Thus, the optimal ICG concentration and laser power density were determined to be 15 μg/mL and 1 W/cm^2^, respectively. PSA-DOX/ICG-Lip (15 μg/mL) irradiated with an 808 nm laser (1 W/cm^2^) reached a temperature of approximately 45 °C (Figure 3C,D), which was sufficient to induce tumor cell necrosis without damaging normal tissues. At the same ICG concentration, PSA-DOX/ICG-Lip exhibited a higher photothermal conversion efficiency than Free ICG, ICG-Lip, and DOX/ICG-Lip (Figure 3E), which was due to the fact that PSA modification reduced the aggregation of ICG molecules in the liposomes and improved the photothermal conversion efficiency. The photothermal stability results showed that the temperature change curves of DOX/ICG-Lip and PSA-DOX/ICG-Lip had no significant changes after five freeze–thaw cycles (Figure 3F,G), indicating good photothermal stability. Thermal imaging analysis (Figure 3H) showed that the temperature of PSA-DOX/ICG-Lip suspension reached approximately 42 °C after 15 min of laser irradiation, which was consistent with the temperature measurement results, confirming the excellent photothermal conversion property of PSA-DOX/ICG-Lip.

### 3.3. In Vitro Anti-Proliferative Effect Study

**Figure 4:**
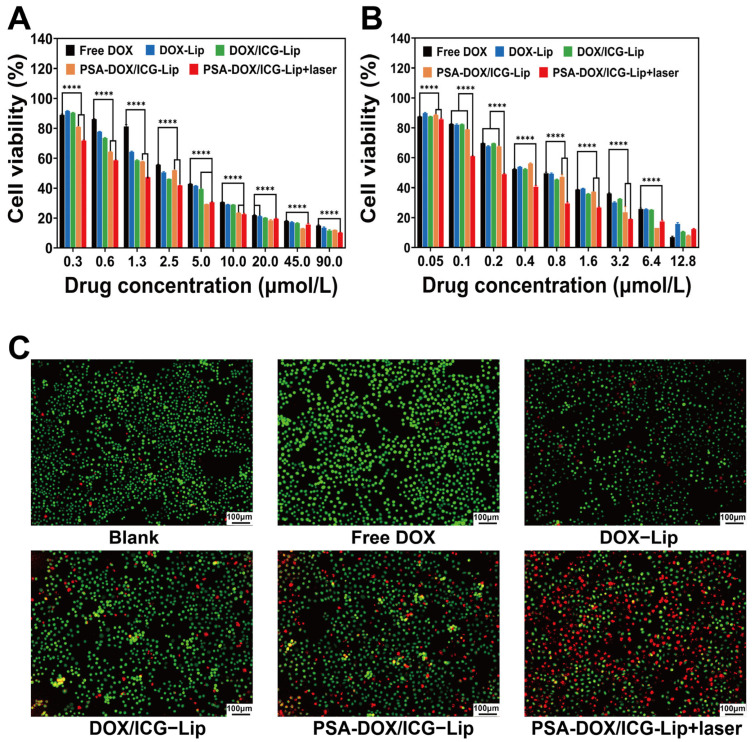
In vitro anti-proliferative effect study. (**A**) Toxic effects of different liposomes on HeLa cells, **** *p* < 0.0001; (**B**) Toxic effects of different liposomes on C33a cells; (**C**) Live/dead staining of HeLa cells treated with different liposomes. In the image, red represents dead cells, and green represents live cells.

The cytotoxicity of different formulations on HeLa and C33a cells with or without 808 nm laser irradiation was evaluated by the CCK-8 assay, and the results are shown in Figure 4A,B. The cell viability of both HeLa and C33a cells decreased with the increase in DOX concentration, and the laser irradiation group exhibited significantly lower cell viability than the non-irradiation group (*p* < 0.01). The anti-proliferative activity of different formulations was in the order of: Free DOX < DOX-Lip < DOX/ICG-Lip < PSA-DOX/ICG-Lip < PSA-DOX/ICG-Lip + laser. The IC_50_ values of different formulations on HeLa and C33a cells are summarized in Table 3. For HeLa cells, the IC_50_ of PSA-DOX/ICG-Lip + laser was 1.25 μM, which was 3.5-fold, 2.6-fold, 2.1-fold, and 1.6-fold lower than that of Free DOX, DOX-Lip, DOX/ICG-Lip, and PSA-DOX/ICG-Lip, respectively. For C33a cells, the IC_50_ of PSA-DOX/ICG-Lip + laser was 0.26 μM, which was 3.2-fold, 3.0-fold, 2.8-fold, and 2.4-fold lower than that of the corresponding formulations, respectively. Calcein-AM/PI double staining results (Figure 4C) showed that the PSA-DOX/ICG-Lip + laser group had the reddest fluorescence (dead cells) and the least green fluorescence (live cells), which was consistent with the CCK-8 assay results, confirming the strongest anti-proliferative effect of PSA-DOX/ICG-Lip combined with laser irradiation on cervical cancer cells.

### 3.4. Cellular Uptake and Neutrophil Targeting

**Figure 5:**
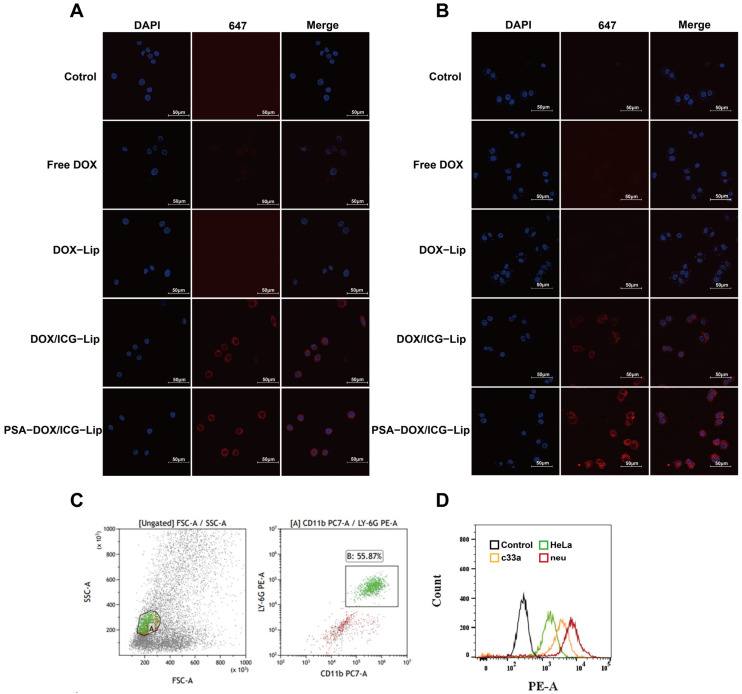
Cell uptake of different liposomes. (**A**) Laser confocal detection of HeLa cell uptake of liposomes. The blue stain marks the cell nuclei; the red light is the fluorescence of ICG; (**B**) Laser confocal detection of C33a cell uptake of liposomes. The blue stain marks the cell nuclei; the red light is the fluorescence of ICG; (**C**) Neutrophil identification by Flow cytometry. The green circle represents CD11b^+^/Ly6G^+^ double-positive cells, i.e., neutrophils; FSC-A (forward scatter): Indicates cell size. SSC-A (side scatter): Indicates intracellular particles or complexity; (**D**) Flow Cytometry Analysis of PSA-DOX/ICG-Lip Targeting Neutrophils.

The cellular uptake efficiency of different formulations by HeLa and C33a cells was evaluated by LCSM, and the results are shown in Figure 5A,B. The fluorescence intensity of DOX in the cells increased in the order of: Free DOX ≈ DOX-Lip < DOX/ICG-Lip < PSA-DOX/ICG-Lip, indicating that PSA modification significantly enhanced the cellular uptake efficiency of liposomes by cervical cancer cells. The neutrophil isolation and identification results (Figure 5C) showed that the purity of isolated mouse neutrophils was 55.87%, which was sufficient for subsequent experiments. Flow cytometry results (Figure 5D) showed that the fluorescence intensity of PSA-DOX/ICG-Lip in neutrophils was significantly higher than that in HeLa and C33a cells (*p* < 0.01), indicating that PSA-DOX/ICG-Lip had a specific targeting ability to neutrophils, which was attributed to the specific binding between PSA and L-selectin on the neutrophil surface.

### 3.5. Intracellular ROS Detection

**Figure 6:**
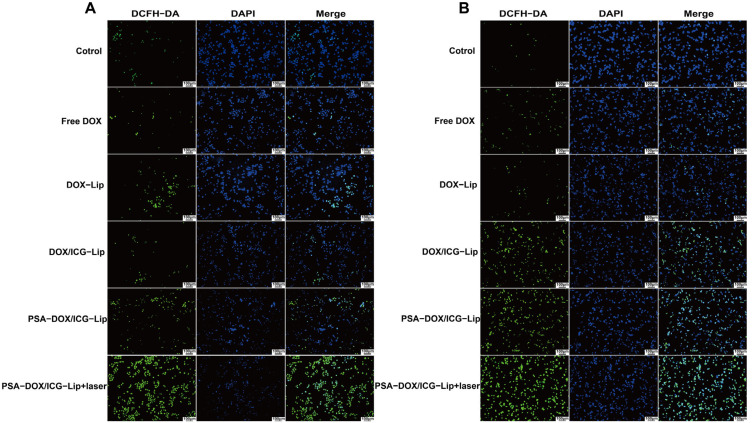
Intracellular ROS Detection. (**A**) Effects of PSA-DOX/ICG-Lip on Intracellular Reactive Oxygen Species Levels in HeLa Cells; (**B**) Effects of PSA-DOX/ICG-Lip on Intracellular Reactive Oxygen Species Levels in C33a Cells. In the figure, green represents ROS, and blue represents the cell nucleus.

The intracellular ROS generation induced by different formulations with or without laser irradiation was evaluated by the DCFH-DA probe, and the results are shown in Figure 6. The fluorescence intensity of DCF (reflecting intracellular ROS levels) in HeLa and C33a cells was the highest in the PSA-DOX/ICG-Lip + laser group, followed by the PSA-DOX/ICG-Lip group, and was the lowest in the Free DOX group. The results indicated that ICG-mediated PTT could significantly induce intracellular ROS generation, and PSA modification enhanced the ROS generation by promoting the cellular uptake of liposomes. The excessive ROS generation could damage the cellular membrane and organelles, induce oxidative stress, and ultimately lead to tumor cell death, which is an important mechanism for the synergistic anti-tumor effect of chemotherapy and PTT.

### 3.6. Cell Scratch Test

**Figure 7:**
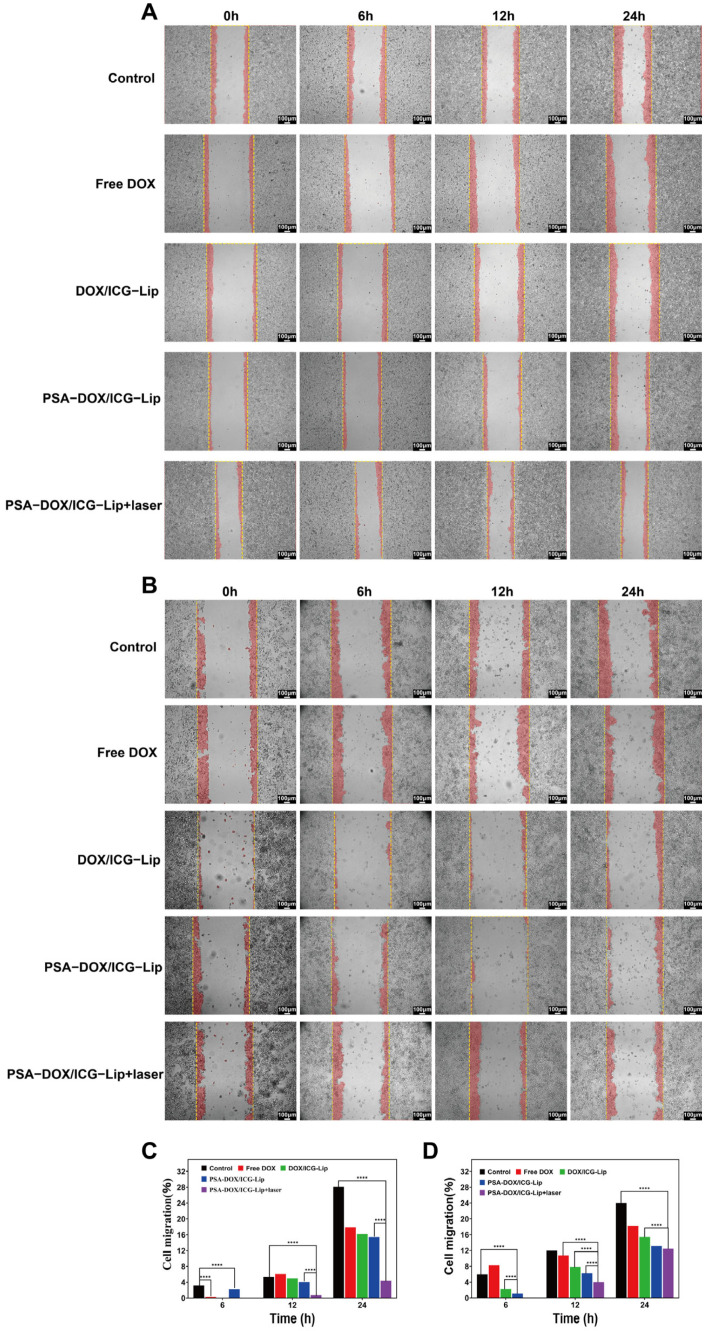
Cell Scratch Test. (**A**) Scratch experiments on HeLa cells by different liposomes; (**B**) Scratch experiments on C33a cells by different liposomes; (**C**) Healing rate of different groups of HeLa cells; (**D**) Healing rate of different groups of C33a cells. **** *p* < 0.0001.

The anti-migration effect of different formulations on HeLa and C33a cells was evaluated by the cell scratch assay, and the results are shown in Figure 7. The wound healing rate of the cells in the drug-treated groups was significantly lower than that in the blank control group (*p* < 0.01), and the laser irradiation group exhibited a lower wound healing rate than the non-irradiation group (*p* < 0.05). The PSA-DOX/ICG-Lip + laser group had the lowest wound healing rate among all groups, with the wound healing rate of HeLa cells and C33a cells less than 20% at 24 h, indicating that PSA-DOX/ICG-Lip combined with laser irradiation could significantly inhibit the migration of cervical cancer cells, which was beneficial for preventing tumor metastasis.

### 3.7. In Vivo Pharmacodynamic Evaluation

**Figure 8:**
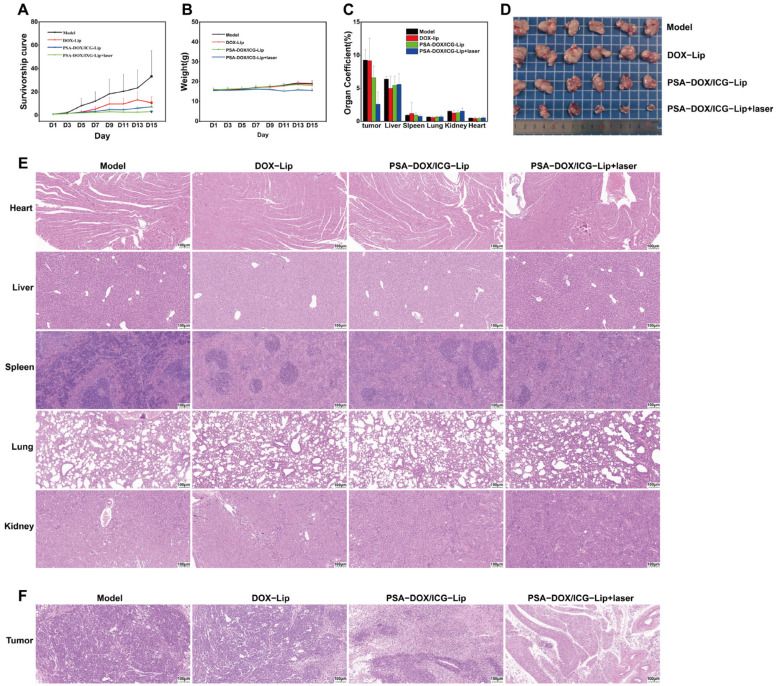
In vivo pharmacodynamic experiments. (**A**) The changes in tumor volume in BABL/C-nu mice over 14 days (*n* = 6, mean ± SD); (**B**) The weight changes in BABL/C-nu mice over 14 days (*n* = 6, mean ± SD); (**C**) The specific gravity of internal organs in BABL/C-nu mice (*n* = 6, mean ± SD); (**D**) Tumor images of BABL/C-nu mice; (**E**) HE staining plots of organs in BABL/C-nu mice; (**F**) HE staining plots of tumors in BABL/C-nu mice. In H&E staining, the cell nuclei appear blue-violet, while the cytoplasm and extracellular matrix appear pink or pale red.

The in vivo anti-tumor efficacy of different formulations was evaluated using a BALB/c-nu nude mouse xenograft model of cervical cancer, and the results are shown in Figure 8 and Table 4. The tumor volume of the negative control group increased rapidly, with the final tumor volume reaching 33.3 times the initial volume. The tumor growth in the drug-treated groups was significantly inhibited, and the anti-tumor efficacy was in the order of: DOX-Lip < PSA-DOX/ICG-Lip < PSA-DOX/ICG-Lip + laser. The final tumor volume of the PSA-DOX/ICG-Lip + laser group was only 3.2 times the initial volume, which was significantly lower than that of the DOX-Lip group (10.7-fold) and PSA-DOX/ICG-Lip group (7.2-fold) (*p* < 0.001). The tumor inhibition rate of the PSA-DOX/ICG-Lip + laser group reached 81.5%, which was significantly higher than that of the PSA-DOX/ICG-Lip group (28.8%) (*p* < 0.001), while the DOX-Lip group had no significant tumor inhibition effect (*p* > 0.05).

The body weight change in the mice during the experiment is shown in Figure 8B. Compared to the initial body weight, the model group gained 22.6% by the end of the experiment, the DOX-Lip group increased by 17.6%, and the PSA-DOX/ICG-Lip group increased by 13.8%, while the PSA-DOX/ICG-Lip + laser group experienced a 0.6% decrease in final body weight; however, this difference was not statistically significant (*p* > 0.05), indicating that this formulation exhibited no significant systemic toxicity. Organ coefficient results (Figure 8C) show that the organ coefficients for the heart, liver, spleen, lungs, kidneys, and tumor in the control group were (0.51 ± 0.06), (6.41 ± 0.37), (0.94 ± 0.09), (0.64 ± 0.06), (1.52 ± 0.15), (9.28 ± 1.58). Compared with the control group, the organ coefficients for the heart (0.62 ± 0.06), lungs (0.78 ± 0.04), and kidneys (1.77 ± 0.08) in the PSA-DOX/ICG-Lip + laser group were slightly higher (*p* < 0.01), which was attributed to transient immune cell infiltration induced by the antitumor immune response.

The H&E staining results of the major organs (heart, liver, spleen, lung, and kidney) of the mice are shown in Figure 8E. No obvious histopathological damage (e.g., inflammation, necrosis, fibrosis) was observed in the major organs of the PSA-DOX/ICG-Lip + laser group, indicating good in vivo biosecurity of the formulation. The H&E staining results of the tumor tissues (Figure 8F) showed that the tumor cells in the negative control group were closely arranged with vigorous proliferation (binucleated and multinucleated cells were observed). In contrast, the tumor tissues in the drug-treated groups exhibited loose arrangement, extensive neutrophil infiltration, and large areas of necrosis, and the PSA-DOX/ICG-Lip + laser group had the most extensive tumor necrosis area, which was consistent with the in vivo anti-tumor efficacy results, confirming the strong synergistic anti-tumor effect of PSA-DOX/ICG-Lip combined with laser irradiation.

## 4. Discussion

In the field of tumor therapy, phototherapy has emerged as a research hotspot for treating malignant tumors due to its high selectivity, effective accumulation at tumor sites, minimal damage to normal tissues, and potential for repeated treatment [26]. This study proposes a PSA-modified targeted delivery strategy aimed at achieving synergistic antitumor effects through the combination of photodynamic therapy (PDT), PTT, and chemotherapy. Liposomes co-loaded with DOX and ICG were prepared using microfluidic technology. TEM revealed that PSA-DOX/ICG-Lip exhibited uniform spherical morphology with an average diameter of approximately 90 nm, a hydrodynamic size comparable to this value, and excellent dispersibility in water. The liposomes exhibited a 90% encapsulation efficiency and 8% drug loading capacity, demonstrating high-efficiency drug encapsulation and delivery capabilities. Beyond morphological characterization, this study further analyzed the optical and chemical properties of PSA-DOX/ICG-Lip. In vitro non-cellular studies of photothermal performance and DOX release behavior demonstrated that, compared to conventional single PTT strategies, PSA-DOX/ICG-Lip achieved tumor thermal ablation at 42 °C while triggering high-level DOX release. Concurrently, it prolonged the half-life of Free ICG, synergistically enhancing therapeutic efficacy. Cellular experiments revealed that PSA-DOX/ICG-Lip exhibited low toxicity toward HeLa and C33a cells under dark conditions but demonstrated significant phototoxicity upon illumination, with IC_50_ values of 1.25 μM and 0.26 μM for HeLa and C33a cells, respectively, indicating its potential for tumor-specific activation. Furthermore, PSA-DOX/ICG-Lip efficiently induced ROS generation within HeLa cells, further validating its photodynamic effects. In vivo pharmacodynamic evaluations demonstrated that the combination of PSA-DOX/ICG-Lip with PTT/PDT treatment significantly enhanced tumor cell death levels and exhibited the strongest tumor growth inhibition. However, it is important to acknowledge that the BALB/c-nu nude mouse model used in this study cannot fully recapitulate the complex stromal components and immune microenvironment of human solid tumors. Further validation using patient-derived xenograft (PDX) or syngeneic models is warranted. In summary, compared with previously reported biomimetic delivery systems, this study integrates PSA-mediated targeted delivery with the ternary synergistic strategy of PTT/PDT/chemotherapy, overcoming the limitations of passive targeting and effectively addressing the constraints of single-drug therapy. PSA can specifically bind to selectins overexpressed on tumor vasculature and cells, conferring active targeting ability, thereby enhancing drug accumulation at the tumor site and significantly improving the anti-tumor therapeutic effect.

## 5. Conclusions

This study successfully constructed a dual-drug delivery system, PSA-DOX/ICG-Lip, using PSA as a targeting molecule. The system was prepared with high encapsulation efficiency and stable physicochemical properties through microfluidic methods and ammonium sulfate gradient methods. Under photothermal therapy, the system can effectively release drugs, significantly enhancing the cytotoxic effect on cervical cancer cells, and has been experimentally verified for its targeting of cancer cells and neutrophils. Overall, this study provides a novel and effective strategy for the treatment of cervical cancer.

## Figures and Tables

**Table 1:** Encapsulation efficiency (EE) and drug loading capacity (LC) of different liposomal formulations (*n* = 3, mean ± SD).

Group		EE (%)	LC (%)
DOX	DOX-Lip	90.53 ± 0.97 ***	8.16 ± 0.09 ***
	DOX/ICG-Lip	90.72 ± 1.13 ***	8.17 ± 0.10 ***
	PSA-DOX/ICG-Lip	96.52 ± 0.43	8.70 ± 0.04
ICG	ICG-Lip	65.20 ± 0.84 ****	0.59 ± 0.01 ****
	DOX/ICG-Lip	80.09 ± 1.41 ***	0.72 ± 0.01 ***
	PSA-DOX/ICG-Lip	90.72 ± 1.10	0.82 ± 0.02

*** *p* < 0.001, **** *p* < 0.0001.

**Table 2:** Particle size, PDI, and zeta potential of different liposomal formulations (*n* = 3, mean ± SD).

Group	Size (nm)	PDI	Zeta Potential (mV)
Blank-Lip	82.62 ± 0.44	0.04 ± 0.03	−9.06 ± 0.47
ICG-Lip	93.05 ± 1.51	0.07 ± 0.01	−11.43 ± 0.76
DOX-Lip	82.22 ± 1.52	0.03 ± 0.01	−10.43 ± 0.31
DOX/ICG-Lip	91.81 ± 0.79	0.05 ± 0.06	−13.17 ± 0.61
PSA-DOX/ICG-Lip	92.68 ± 1.14	0.04 ± 0.00	−9.66 ± 0.46

**Table 3:** IC_50_ of different Liposomes on HeLa cells and C33a cells.

IC_50_ (μM)	HeLa	C33a
Free DOX	4.34	0.84
DOX-Lip	3.24	0.79
DOX/ICG-Lip	2.68	0.72
PSA-DOX/ICG-Lip (−)	2.00	0.63
PSA-DOX/ICG-Lip (+)	1.25	0.26

**Table 4:** The growth and inhibition rate of tumors in BABL/C-nu mice (*n* = 6, mean ± SD).

%	DOX-Lip	PSA-DOX/ICG-Lip	PSA-DOX/ICG-Lip + Laser
RTV	32.3 ± 15.3	21.8 ± 10.3	9.5 ± 3.3
TIR	−14.9 ± 23.0	28.8 ± 16.6	81.5 ± 11.0

## Data Availability

Most included studies are publicly available via open access on journal websites. Additional data and code are available upon request for privacy reasons.

## Abbreviations

The following abbreviations are used in this manuscript:

PSA	Polysialic Acid
DOX	Doxorubicin
ICG	Indocyanine Green
PDI	Polydispersity Index
NIR	Near-Infrared Spectrum
DPPC	Dipalmitoyl Phosphatidylcholine
HSPC	Hydrogenated Soy Phospholipids
PBS	Phosphate-Buffered Saline
TEM	Transmission Electron Microscope
ROS	Reactive Oxygen Species
DCFH-DA	2′,7′ -Dichlorofluorescein Diacetate
DAPI	4′,6-Diamidino-2′-Phenylindole
TANs	Tumor-associated Neutrophils
